# Liver organoids: modelling complexity in homeostasis and disease

**DOI:** 10.1002/1873-3468.70432

**Published:** 2026-09-03

**Authors:** Anna M. Dowbaj, Meritxell Huch

**Affiliations:** ^1^ School of Life Sciences and Center for Organoid Systems (COS) Technical University of Munich Germany; ^2^ Max‐Planck‐Institute of Molecular Cell Biology and Genetics Dresden Germany

**Keywords:** liver assembloids, liver organoids, modelling homeostasis and disease, tissue architecture

## Abstract

Liver is a complex organ, capable of incredible regenerative capacity in the mammalian body. In addition to well‐known hepatocytes, liver is composed of a complex cell microenvironment, with each cell type contributing to homeostasis, regeneration and disease. Modelling of the liver *in vitro* has been hampered by 2D cell lines being too simple and whole animal models too complex to underpin the mechanisms of liver biology. Liver organoids are emerging as a great tool to study liver (patho)physiology. Increasing in complexity, organoids have successfully been used to understand the cellular interplay in genetic and metabolic liver diseases, as well as liver cancer. This review will discuss the advancement in modelling cell complexity, maturation and liver architecture in a Petri dish. It will also provide a state‐of‐the‐art perspective of what is still lacking and how the models can be further improved. Finally, we will conclude with our personal vision of where the field is going.

## Graphical abstract

Studying liver *in vitro* has been challenging because simple 2D cell cultures fail to capture liver's cellular and architectural complexity. To bridge this gap, scientists increasingly use organoids, 3D liver models which better mimic liver composition and function. This review examines recent advances in liver organoid complexity and realism, discusses the remaining limitations and highlights how future developments could significantly enhance organoid research utility.

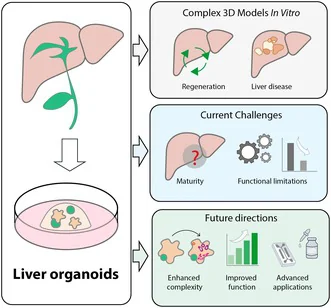

## Abbreviations

BMP bone morphogenic protein

CAF cancer‐associated fibroblast

cAMP cyclic adenosine monophosphate

CC cholangiocarcinoma

CHC combined HCC/CC cancer

CholOrgs cholangiocyte organoids

DE definitive endoderm

DILI drug‐induced liver injury

EC endothelial cells

EGF epidermal growth factor

ESC embryonic stem cells

FAH fumarylacetoacetate hydrolase

FGF fibroblast growth factor

FXR farnesoid X receptor

HBV hepatitis B virus

HCC hepatocellular carcinoma

HepOrgs hepatocyte organoids

HHO human hepatocyte organoids

HSC hepatic stellate cells

HUVEC human umbilical vein endothelial cells

IL interleukin

iPSC induced pluripotent stem cells

iPSC‐HE induced pluripotent stem‐cell hepatic endoderm

iPSC‐LB induced pluripotent stem‐cell liver bud

KO knockout

LGR5 leucine‐rich repeat‐containing G‐protein coupled receptor 5

LSEC liver sinusoidal endothelial cell

MaguOs macrophage‐augmented organoids

MSC mesenchymal stem cells

PDMS polydimethylsiloxane

PHH primary human hepatocyte

PLC primary liver cancer

scRNAseq single cell RNA sequencing

STAT3 Signal Transducer and Activator of Transcription 3

TGF transforming growth factor

TNF tumour necrosis factor

Liver is the biggest solid internal organ in our body, responsible for many vital functions, such as detoxifying blood, storing energy and processing nutrients. It is also an organ capable of regrowing after 70% removal or regenerating surprisingly well following injury [1, 2]. It is not fully understood why sometimes this impressive regenerative capacity fails, and liver progresses to a myriad of diseases—ranging from fibrosis to cancer. As every organ, liver is composed of a complex cell microenvironment. Liver parenchyma is formed by hepatocytes, big and often multinucleated cells with an unusual polarity. Their apical sides form a series of small but interconnected channels, called bile canaliculi. Hepatocytes are the cells which usually regenerate the liver, but they also produce bile, detoxify the blood and perform enzymatic and storage functions of this organ [1, 2]. Cholangiocytes, also known as biliary epithelial cells or ductal cells, are another liver epithelial cell type, which like hepatocytes during development are derived from endoderm. Cholangiocytes collect bile produced by hepatocytes and transport it through the liver to extrahepatic ducts, gallbladder and the digestive tract. Cholangiocytes can also regenerate the liver in the process known as ductular reaction [3, 4, 5]. Hepatocytes and cholangiocytes form two tubular networks, which connect in a structure known as Canal of Hering [6].

### Liver microenvironment

Liver epithelial cell types exhibit significant heterogeneity within their populations [7]. Hepatocyte heterogeneity and division of labour is underlined by the gradients of nutrients and oxygen in the liver lobule—also known as liver zonation. The periportal part of the liver lobule (Zone 1) is rich in oxygen and nutrients, which decrease through the midlobular region (Zone 2) towards the central vein (Zone 3) with the blood flow direction [7, 8, 9]. Hepatocytes in different zones vary in gene expression, and perform different functions, for example portal hepatocytes are responsible for urea metabolism, while central vein hepatocytes are responsible for glutamine synthesis activity. Cholangiocytes were formerly divided according to their location in the tissue whether intrahepatic or extrahepatic, as well as from the size of branches they originated from [10]. Recent studies showed much greater nuance in the transcriptional profiles and morphology of these heterogenous cells [11, 12, 13]. Liver zonation corresponds to compartmentalization of liver disease initiation, with for example acetaminophen (paracetamol) and ethanol toxicity arising in the pericentral region, while biliary disorders being restricted to the periportal region [14, 15].

In addition to epithelial cells, the liver contains several nonparenchymal cell types derived from mesoderm during development, which are divided into mesenchymal, endothelial and immune populations. Mesenchymal cell populations consist of hepatic stellate cells (HSC), portal fibroblasts (residing in the periportal region) and vascular smooth muscle cells around the hepatic artery, with mesothelial cells and capsular fibroblasts in the liver capsule [16, 17, 18]. Endothelial cells (ECs) are divided into the liver sinusoidal endothelial cells (LSECs), which are zonated and have a fenestrated morphology and permeable phenotype, and portal, central and arterial vein endothelial cells, which line the portal and hepatic veins and the hepatic artery, respectively [18, 19, 20]. Once again, immune cells in the liver consist of several heterogenous populations, such as Kupffer cells (liver macrophages), monocytes, dendritic cells, NK cells, T cells, B cells and neutrophils [18, 21, 22, 23]. The intricate network and tissue architecture result in complex cellular interactions that reflect the complex physiology and pathophysiology of the tissue.

### Liver organoids

For decades, the study of the liver and liver diseases has been only possible *in vivo*, in animal models. Cultured hepatocytes in 2D, in sandwich culture or as spheroids (aggregates of hepatocytes in suspension), retain hepatocyte polarity. However, their lack of expandability in culture and rapid loss of hepatic function render them unsuitable to study phenotypes that extend beyond a few days, including those relevant for the emergence of disease states. Like primary hepatocytes, liver cancer cell lines have been instrumental to elucidate many aspects of liver physiology. However, these models fail to capture the epithelial and nonparenchymal cell heterogeneity of the liver. Organoids, tiny liver avatars also sometimes called ‘liver in a dish’, are a step towards modelling the complex cell composition and organ architecture in 3D. Here, we adopt the definition of organoids proposed by Huch and Koo [24], Sato and Clevers, and Lancaster and Knoblich (see Box 1). In this definition, an organoid is a 3D culture derived from a stem cell [induced pluripotent stem cell (iPSC), embryonic stem cell (ESC), tissue‐resident stem cell or progenitor], a differentiated cell or a fetal progenitor that, when exposed to appropriate growth factors and often a 3D extracellular matrix, self‐organizes into structures that resemble the organ of origin and exhibit some of its functions. In the specific case of the liver, liver organoids grow into complex multicellular structures, can produce bile and metabolise nutrients to levels comparable with freshly isolated liver cells. Additionally, the concept of assembloids [25, 26] has also recently been adapted for the studies of the liver [27, 28], and is understood here as organoids generated from multiple primary or organoid cell types, which self‐organise spatially.

Box 1 **Organoid definitions**

Organoid definition according to Huch and Koo: They define an organoid as a 3D structure in which cells spontaneously self‐organise into progenitors and differentiated functional cell types that resemble the original organ and recapitulate some of its functions. The first review to separate both types of organoid models according to the cell of origin being embryonic stem cells (ESC)/induced pluripotent stem cells (iPSC) or resident adult tissue (ASC) [24].

Organoid definition according to Lancaster and Knoblich: definition proposed is based on three essential requirements that need to be fulfilled: first, an organoid must contain more than one cell type of the organ it models; second, it must recapitulate some of the specific functions of that organ; and third, the cells should have a similar spatial organization as the organ [29].

Organoid definition according to Clevers: a 3D structure grown from stem cells and consisting of organ‐specific cell types that self‐organizes through cell sorting and spatially restricted lineage commitment [30].

In this review, we will describe all relevant literature which conforms to the above definition for liver organoids, as opposed to spheroids or structures formed by immortalized cell lines, sometimes wrongly annotated as organoids. We outline both mouse and human liver organoid models (for other species, please see [31]). We will first provide a short historical perspective on organoid models, followed by the characterization of the first tissue‐derived and iPSC/ESC‐derived liver organoid models. We will follow with sections delving deeper into describing the different levels of achieving complexity: (1) increasing epithelial cell heterogeneity and expansion, (2) incorporation of the stem‐cell niche and nonparenchymal cell types, and (3) achieving tissue architecture reconstruction (modelling biliary tree, bile canaliculi, blood vessel structures and zonation) while maintaining functional maturation in the organoid models. Lastly, we will outline current efforts of increasing complexity in the context of liver cancer modelling, describing current organoid models of liver cancer and the efforts of including tumour microenvironment cells in the organoid models. In the last section, we will summarise the main challenges faced today by the liver organoid field.

## Achieving expansion and heterogeneity of epithelial cells

### Historical perspective

The first citation with the word ‘organoid’ dates back to 1946, when scientists used it to describe a teratoma growing in a newborn [32]. After that, scientists termed organoids as any 3D type of primary culture that would grow *in vitro* [33]. In the case of the liver, in 2001, George K. Michalopoulos cultured digested rat liver tissue in roller bottles coated with collagen and supplemented with their previously defined medium for 2D hepatocytes [34], and described the formation of 3D structures with histological resemblance to the tissue [35]. Despite retaining some of the tissue features, these cultures do not match the modern definition of organoids we adhere to here (Box 1). The definition in Box 1 is distinct from spheroids, understood here as 3D cell aggregates, often composed of primary hepatocytes or from immortalized cell lines. The differences between organoids and these distinct cellular models have been extensively reviewed elsewhere [36]. The rediscovery of organoids derived from primary tissue, as we understand them today, can be traced to a landmark 2009 study from the Clevers Lab [37]. In that work, the authors showed that Lgr5^+^ intestinal stem cells could form intestinal organoids when embedded in the extracellular matrix mix Matrigel and supplied with cytokines that replaced the stem‐cell niche signals in the culture medium [37]. By supplementing this intestinal medium with FGF10 and WNT ligand, both important to maintain stomach epithelium, gastric organoids that retained not only Lgr5^+^ gastric stem cells but also their differentiated progeny could be readily generated [38].

### Tissue‐derived liver organoids

In 2013, the first liver organoids derived from mouse tissue were established [39]. Following on from the seminal work of Michalopoulos [34, 35], Lemaigre [40, 41], Zaret [42] and Nusse [43] that had identified the importance of HGF, EGF, FGF, Notch and WNT signalling in liver development and repair, combined with the expertise from developing gastric organoids [38], Huch et al. identified culture conditions to grow the liver cells as the first liver organoids (Fig. 1, top). This organoid system allowed the long‐term culture not only of primary ducts from healthy liver but also of ductal cells from damaged livers. The cells could be clonally expanded and, when cultured in conditions of Notch inhibition supplemented with dexamethasone, the cells were able to differentiate into hepatocyte‐like cells that secreted albumin, were able to rescue the phenotype of FAH−/− mutant mice upon transplantation, and differentiated into fully functional hepatocytes *in vivo* [39]. Following this pioneering work using mouse tissue, Huch et al. set to translate this approach into human cells. Interestingly, the translation of mouse model to the human system was not immediate and required several iterations to the protocol. First, the authors showed that, unlike in mice, human liver cells exhibit high levels of TGFβ signalling, and that inhibition of this pathway is necessary—though not sufficient—for their expansion [44]. By integrating this knowledge with findings from mouse models showing that cAMP activation is important for ductal cell proliferation *in vivo*, Huch *et al*. identified culture conditions that support the long‐term expansion of human liver cells *in vitro* [44]. Because, like the mouse model, the authors could generate millions of cells in culture and differentiate them into hepatocyte‐like cells, the researchers were able to transplant these cells into immunodeficient mice and observe their engraftment, differentiation into mature hepatocytes and secretion of human albumin into the mouse bloodstream [44]. Interestingly, by performing whole genome sequencing analysis in clonally derived human liver organoid cultures, the authors confirmed that the expanded cells retain their genetic stability over time [44]. This work provided the first evidence that human primary not‐transformed cells can be serially expanded *in vitro*, defying the so‐called Hayflick limit of 60 cell divisions [45, 46, 47]. Following this observation, other reports have shown similar genetic stability in other *in vitro* organoid models, including the prostate [48], pancreas [49] and gut [50].

**Fig. 1:**
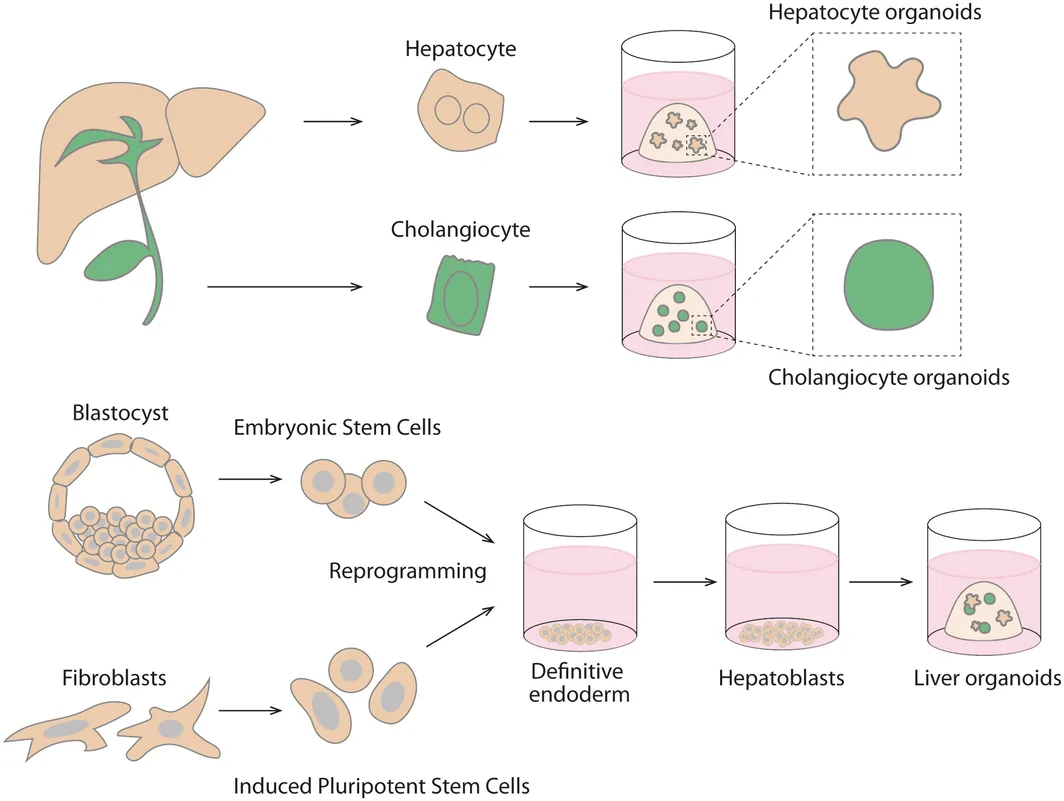
Schematic showing the generation of tissue‐derived or ESC/iPSC liver organoids. Tissue‐derived liver organoids are generated from a liver biopsy, which is usually further processed to obtain pure or cell‐enriched fractions of cholangiocytes or hepatocytes. Both of those cell types can form organoids when cultured in extracellular matrix with the right growth factors. Cholangiocytes grow as cystic and hepatocytes as bubbly/grape‐like structures. For the ESC/iPSC, the cells are obtained from embryo or through reprogramming, respectively. The cells are differentiated first to definitive endoderm, then to hepatoblasts and finally can be directed to either cholangiocyte or hepatocyte lineage, as needed, by a combination of growth factors and extracellular matrix embedding. ESC, embryonic stem cells; iPSC, induced pluripotent stem cells.

Back in 2013, Huch et al. interpreted the ability of these first liver organoids to self‐propagate from single (clonal) cells and differentiate towards the hepatocyte lineage as evidence that they were derived from stem cells that would be activated upon damage [39]. However, follow up studies by the Huch Lab [51] and other laboratories, including the Clevers [52], Grompe [53], Camargo [54] and Tchorz [55] have shown that the cell of origin of these liver organoid cultures is a bona‐fide differentiated ductal cell that de‐differentiates, acquires progenitor features *in vitro* and starts to proliferate (Fig. 1, top). That resulted in later renaming of this culture system in a consensus paper by Marsee *et al*. in 2021, as ‘intrahepatic cholangiocyte organoids, or (ICOs)’ [56] as opposed to liver organoids, to facilitate their distinction from organoids derived from hepatocyte cells [52, 57] or extrahepatic cholangiocytes (ECOs) [58]. Therefore, from hereon we will term these first liver organoids as CholOrgs, to fit this renaming and change in nomenclature.

While these first liver organoids (CholOrgs) were able to model hepatocytes as organoids *in vitro* for the first time, they lacked full hepatocyte maturity and typical hepatocyte polarity. CholOrgs closely resembled key features of cholangiocytes in terms of gene expression and functionality, as well as mimicked the response of cholangiocytes to liver damage. CholOrgs grew as spherical one cell layer structures with a lumen [39, 44], in contrast to the tubes seen *in vivo*. Similarly to hepatocytes, however, cholangiocytes missed some of the differentiation markers and differentiation states of mature cells seen in the homeostatic cholangiocytes *in vivo*, as expected in a proliferating culture. This lack of heterogeneity was not fully appreciated at the time before the widespread use of scRNAseq to characterise cell populations. Recently, by profiling these cultures at the single cell level, the Huch Lab has found that these first organoids are highly homogenous. By refining the culture conditions, the authors have been able to upgrade the CholOrgs system to capture the ductal cell state heterogeneity in a dish, including the three major liver ductal cell fates: cholangiocyte‐, bipotent‐ and hepatocyte‐like fates [59]. Thus, this refined system captures the heterogeneity of the ductal epithelium *in vitro*, reflecting its *in vivo* complexity as defined by scRNAseq (Fig. 2) [13, 60].

**Fig. 2:**
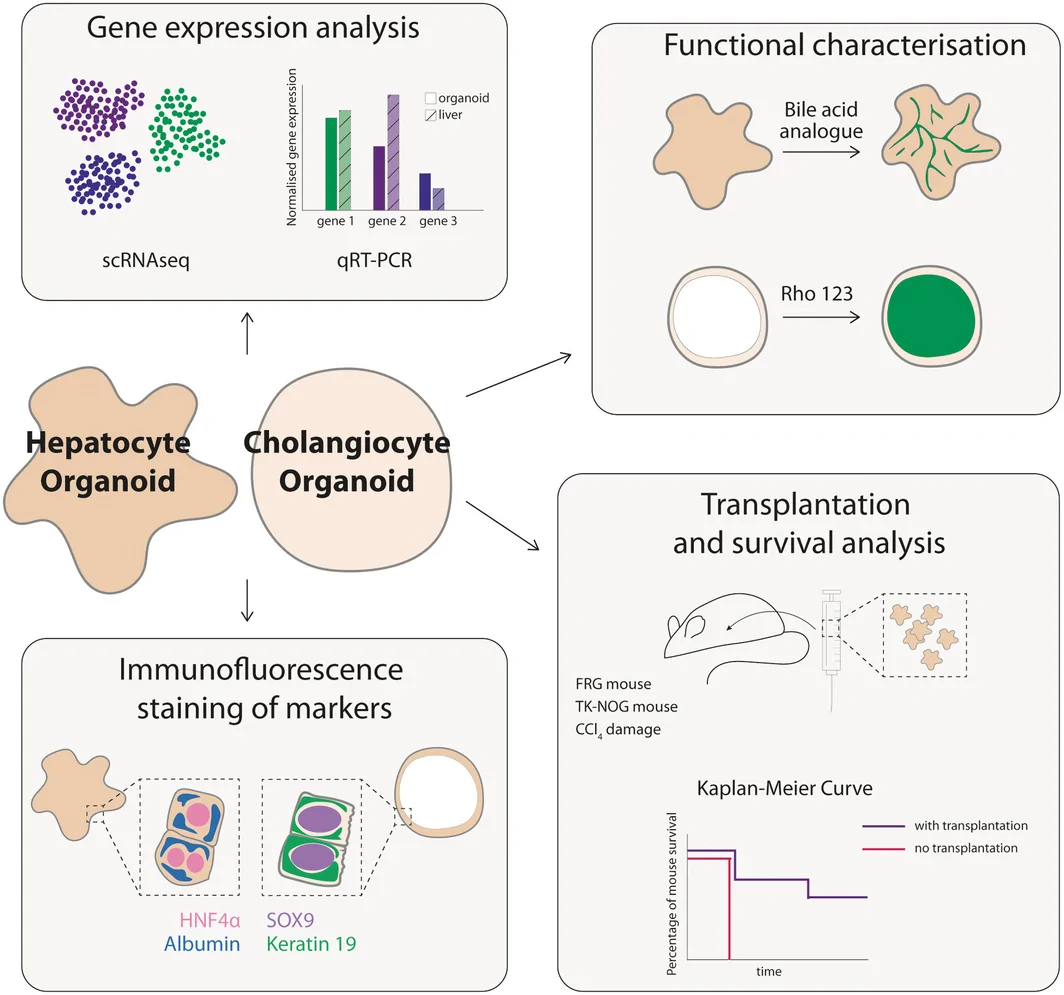
Typical investigation of hepatocyte functionality *in vitro*. Liver organoids are usually investigated by a combination of different methods, which can include gene expression analysis (e.g., qRT‐PCR, scRNAseq), immunofluorescence staining for key identity markers (e.g., HNF4α, Albumin for hepatocytes; SOX9, Keratin 19 for cholangiocytes), functional assays (e.g., fluorescence bile acid transport in hepatocyte organoid bile canaliculi; Rhodamine 123 transport in cholangiocyte organoids), and finally transplantation into a mouse model of liver damage as an ultimate proof of functionality. qRT‐PCR, quantitative real time polymerase chain reaction; scRNAseq, single cell RNA sequencing; Rho 123, Rhodamine 123; FRG mice, Fah knockout mice lacking the genes for Rag‐2 and the common gamma chain of the interleukin receptor; TK‐NOG mice, super immunodeficient NOG mouse with transgenic expression of thymidine kinase under control of liver‐restricted albumin promoter; CCl_4_, carbon tetrachloride.

Soon after this first work on mouse and human liver organoids, the protocol was adopted by many other laboratories to grow other segments of the biliary epithelium. By adding Noggin (BMP inhibitor), Lugli et al. were able to expand gallbladder cells as extrahepatic organoids, while the addition of DKK‐1 (Dickkopf‐1, a secreted WNT inhibitor) to the Huch organoid protocol allowed the expansion of extrahepatic cholangiocytes as organoids [58, 61]. Similarly to the intrahepatic organoids, the extrahepatic organoids exhibited gene expression and functions as those *in vivo*. In follow‐up studies, the Vallier Lab expanded on this research, presenting a comparative study deriving ductal cell organoids from different organ locations: common bile duct, pancreatic duct and gallbladder [62], suggesting that cholangiocytes from different organ locations respond differently to canonical WNT signalling. The same group later demonstrated that biliary epithelial cells from different tissue origins, when cultured as organoids *in vitro*, lose their tissue‐specific characteristics and converge on a similar transcriptional profile, in contrast to the diverse cholangiocyte populations observed *in vivo*. Expanded as organoids, gallbladder cholangiocytes were able to repair intrahepatic bile duct damage when transplanted into mice [63].

While adapting the Huch 2015 protocol for generating liver organoids successfully allowed the establishment of organoids from various ductal populations—including intrahepatic and extrahepatic ducts, gallbladder, and even human pancreatic ducts [49, 64]—establishing organoids from hepatocytes proved more challenging. Zhang et al. opted for an approach of first expanding primary human hepatocytes (Proli‐HH) in hypoxic conditions in 2D, later aggregated them into spheroids, and finally induced differentiation [65], using the media described previously for the human cholangiocyte differentiation into hepatocytes [44]. While these structures were more spheroid than organoids, by our definition above, the authors succeeded in generating an expanded population of 2D hepatocytes with bi‐phenotypic characteristics—that is, expressing both progenitor and hepatocyte markers while lacking cholangiocyte markers—although they did not exhibit the typical 3D hepatocyte polarity. The same year, the first manuscripts which described derivation of organoids originating from mouse and human fetal hepatocytes rather than cholangiocytes were published back‐to‐back in Cell by the Nusse and the Clevers Labs [52, 57] (Fig. 1, top). The authors used different strategies to induce proliferation of murine hepatocyte organoids (HepOrgs) in culture and coerce their further differentiation. Peng et al. used inflammatory signalling by addition of TNFα for proliferation stage, and two differentiation strategies (EGF‐ or WNT‐driven), both with addition of dexamethasone, to induce periportal and pericentral phenotypes of hepatocytes [57]. The study by Hu et al. [52] used parallel WNT agonists, EGF‐FGF7‐FGF10‐HGF addition and TGFβ inhibition to promote hepatocyte proliferation. While the murine hepatocyte organoids could be grown and propagated in culture for several months, the human adult‐derived organoids did not expand. However, the authors succeeded in culturing human fetal HepOrgs, which retained gene expression of human hepatocytes. Additionally, both studies also showed successful HepOrgs engraftment into mouse models. Recently, Hendriks et al. improved the protocol of human fetal HepOrgs culture by increasing proliferation and repressing lipid metabolism using IL‐6 inflammatory cytokine and FXR ligands [66]. The same improved media was applied to adult primary human hepatocytes (PHH), but the organoids showed dysbiosis of lipid metabolism, accumulated lipids and stopped growing. The authors did succeed to expand postnatal donor organoids (0.3–1.7 years) by addition of IL6, FXR ligand and Noggin.

Parallel efforts have also been made to explore the potential of hepatoblasts to form organoids. The Huch Lab isolated mouse LGR5+ positive hepatoblasts and showed that these can form embryonic liver organoids, which retained their bipotent capacity, forming cholangiocyte or hepatocyte organoids depending on the medium conditions [67]. The authors revealed a previously unrecognized early commitment of hepatoblasts to the cholangiocyte fate and, through clonal lineage tracing, showed that a subpopulation of LGR5^+^ hepatoblasts was genuinely bipotent, generating both cholangiocyte and hepatocyte clones that persisted for the entire mouse lifespan. Similarly, the Vallier Lab generated human hepatoblast organoids from EpCAM+ human fetal liver cells, which retained human LGR5+ hepatoblasts in culture and upon Oncostatin M treatment transcriptionally shifted towards immature hepatocyte fate [68]. Interestingly, all hepatocyte organoids presented a different morphology to the cystic cholangiocyte organoids, with a bubbly, grape‐like appearance of the structures [52, 57, 66, 67].

Propagating adult PHH long term has long remained an enigma—which is quite paradoxical considering that one of the most successful regenerators in the human body failed to grow *in vitro*. Recently, two groups achieved long‐term PHH expansion [28, 69] (Fig. 1, top). First, a study from Sato's Lab [69] used commercially available donor‐derived frozen hepatocytes and cultured them in a medium that combined WNT and STAT3 activation, the latter achieved through Oncostatin M, to induce long‐term culture of hepatocytes as functional human hepatocyte organoids (HHO). Those human HHO could be expanded from FACS‐sorted single cells using ASGR1 gene as a marker for hepatocyte population. Interestingly, the authors found that cells with high granularity (high backward side scatter) present higher organoid formation efficiency, which varied depending on the donor. The authors introduced two different differentiation media, one composed of DAPT (N‐[N‐(3,5‐Difluorphenacetyl)‐L‐alanyl]‐S‐phenylglycin‐tert‐butylester; γ‐secretase inhibitor—Notch pathway inhibitor) and a hormone combination (growth hormone, prolactin and cortisol), with or without WNT ligands to induce (or not) central vein zonation signature. Differentiated organoids showed hepatocyte function with bile hepatocyte‐specific lipid and protein production as well as extensive drug metabolism capabilities. Second, a complementary study from the Huch Lab using patient‐derived fresh isolated hepatocytes from multiple individuals (> 28 patients) also showed long‐term expansion of hepatocytes as hepatocyte organoids, when cultures were treated with high WNT and YAP activation (h‐HepOrgs) [28]. Upon growth factor removal, these human HepOrgs acquired mature expression and function resembling freshly isolated hepatocytes, and even outperforming gold‐standard monolayer hepatocytes in drug detoxifying functions. Interestingly, these patient‐derived h‐HepOrgs preserved patient‐to‐patient specific gene signatures as well as presented variation in their bile canaliculi structures, suggesting that when grown as organoids, hepatocytes can capture patient‐to‐patient variation at both gene expression as well as architectural level (Fig. 2). Both models could also be successfully xenotransplanted into mice with liver damage [28, 69], underscoring their potential for cell therapy approaches.

### iPSC and ESC‐derived organoids

For the formation of iPSC‐derived liver organoids, the cells are first differentiated towards definitive endoderm (DE), followed by foregut or hepatic endoderm, which already contain hepatic precursors (Fig. 1, bottom). These hepatic precursors can differentiate both towards a hepatocyte or a cholangiocyte lineage [70]. The first iPSC‐derived 3D model that showed self‐organization was published by Takebe *et al*. in 2013 and named ‘liver bud’—iPSC‐LB [71]. This, in our understanding, fulfils the definition of organoid as we define here, and therefore, we have called them ‘liver bud organoids’. The authors induced hepatic differentiation in iPSCs and combined the hepatocyte‐induced cells (iPSC‐HEs, hepatic endoderm) with human umbilical vein endothelial cells (HUVEC) and human mesenchymal stem cells (MSC), subsequently plating them on a solidified Matrigel to induce the further maturation of hepatocytes. The cells assembled into a 3D structure on the Matrigel layer through MSC contraction. The hepatocyte‐like cells, mesenchyme and endothelium were distributed throughout the structure. The structure resembled a liver during developmental stages, with hepatoblasts‐like cells remaining immature—proliferating and expressing alpha‐fetoprotein (AFP). Remarkably, the iPSC‐LB engrafted upon transplantation in vascularized niches in mice, showed connection to the mouse vasculature and rescued a damage‐induced liver failure mouse model [71]. The same contraction principle was applied to model other organs buds: kidney, pancreas, intestine, heart, lung and brain [72]. Later, the lead authors improved this system, so that the iPSC‐LB could be produced at large scale, now based entirely from iPSC‐differentiated cells: (1) hepatic endoderm cells, (2) endothelium and (3) septum transversum‐like mesenchyme, able to condense the iPSC‐LB structure [73]. These organoids showed an increased level of functionality compared with the previous iPSC model and hepatocytes in 2D, but none of the models explored hepatocyte zonation in detail. Importantly, the authors showed that they could produce structures with 10^8^ cells, which is considered a critical mass for human transplantation.

Parallel efforts have focused on generating cholangiocytes from iPSCs. The Vallier Lab presented a serum‐free differentiation protocol of human iPSC cells into cholangiocyte‐like cells [74], while the Ghanekar Lab used a combination of growth factors and co‐culture with a stromal feeder cell line to achieve cholangiocyte fate from predifferentiated iPSC hepatoblasts [75]. Both studies demonstrated cholangiocyte marker gene expression on RNA and protein level, cholangiocyte‐specific functionality dependent on transporters and modelling of cholangiocyte‐specific disease phenotypes. Interestingly, as for the adult tissue‐derived cholangiocyte organoids—the iPSC‐derived cholangiocyte organoids also grew as hollow cysts, in contrast to liver bud organoids which grew as more condensed structures. Following on from the iPSC organoids, Wang et al. reported derivation of human ESC‐differentiated liver organoids in serum‐free feeder‐free media [76]. These ESC‐organoids could be directed towards either hepatocyte or cholangiocyte fate. However, zonation was not explored in the somewhat immature cell structures, even though the authors demonstrated some functionality and ability to repopulate both epithelial populations *in vivo* when transplanted in mice.

Although in the early studies, the heterogeneity within the epithelial populations was suggested by the varied marker staining between cells—it was not explored in detail. The first study to systematically compare liver bud organoids to the adult and fetal scRNAseq data found striking similarities of iPSC‐derived organoids to the fetal datasets, hinting at immature and embryonic‐like phenotype of these structures [77]. Several groups strived to further mature iPSC‐derived liver buds. In a study by Wang et al., the authors describe a perfusable polydimethylsiloxane (PDMS) micropillar chip system for improved human iPSC‐derived organoids generation, with improved cholangiocyte and hepatocyte marker expression [78]. The cells were seeded as single cells in the device and differentiated using a changing media regime without embedding into ECM. These organoids achieved a bubble‐like morphology by day 20 of culture and could be maintained for up to 1 month [78]. Microfluidics was also applied in another study where authors described human iPSC‐derived organoids in a PDMS microfluidic device and in synthetic temperature‐responsive hydrogel, achieving a degree of hepatocyte maturation [79]. Using the differentiation media from tissue‐derived cholangiocyte organoids [44] on iPSC or ESC‐derived cholangiocytes, further supplemented with nifedipine to promote cytochrome activity, Mun et al. attempted to improve hepatocyte maturity in structures—however, the organoids remained cystic and lacked typical hepatocyte polarity or zonation signatures [80].

## Improving complexity of the stem‐cell niche

### Tissue‐derived organoids

Adult tissue‐derived organoids are characterized by higher maturation compared with the iPSC counterparts as they originate from differentiated or partially differentiated organs rather than an induced developmental trajectory. However, until recently adult liver tissue‐derived organoid models contained only epithelial cells—either cholangiocytes or hepatocytes. Early attempts to introduce niche cells were produced by the hanging drop method, where cryopreserved PHH were combined with commercially acquired Kupffer and endothelial cells, forming a spheroid containing three cell types [81]. Another early approach employing bioprinting was also used, mixing cryopreserved PHH with 2 commercial nonparenchymal cells—HSC and HUVECs [82]. Both studies aimed at better modelling of drug‐induced liver injury (DILI). However, the structures contained mostly hepatocytes with nonparenchymal cells interspersed within the cell mass and failed to reproduce and capture the specific tissue architecture and cell interactions seen in liver tissue. In addition, the formed structures could not be expanded or maintained long term.

More recently, the field of adult stem cell‐derived organoids is striving towards including niche cells into the organoid culture. The first notable example, presented by the Huch Lab, was an introduction of primary portal fibroblasts to CholOrgs culture [83]. When added directly to the culture, the authors observed separation of portal mesenchyme from CholOrgs. The authors were able to achieve contact and growth of the two cell types together using microfluidics or aggregation on a 2D Matrigel layer, which forced the cells into close proximity. The structures better resembled tissue cell arrangement, with cholangiocytes surrounded by portal mesenchyme and in direct contact through the basal cell side with portal mesenchymal cells, resembling the cellular arrangement of the *in vivo* tissue. This improved organoid model led to an interesting discovery and reconciliation of liver homeostasis with cholangiocyte‐driven regeneration, whereby the mesenchyme was controlling the proliferative response of the epithelium: inhibitory when being in direct contact or stimulating cholangiocyte growth by paracrine cytokine signalling [83]. In a more recent subsequent study, the same Huch group leveraged this knowledge to generate a more complex model containing portal fibroblasts, cholangiocytes and an improved hepatocyte organoid model—all derived from murine adult tissues—to generate ‘liver periportal assembloid’ [27]. To generate mouse periportal assembloids, Dowbaj et al. utilized a ‘self‐assembled LEGO’ approach, where they combined individually expanded mouse portal fibroblasts and mouse cholangiocytes derived from cholangiocyte organoids with expanded hepatocyte organoids. For that, the authors first improved the existing adult hepatocyte organoid model from the Clevers Lab [52] by supplementing culture conditions from their previous mouse hepatoblast organoid model [67] with WNT ligand. This allowed the authors to obtain organoids of sufficient size and containing a physiological bile canaliculi network to attempt incorporating additional cells. Then, the authors assembled these hepatocyte organoids with cholangiocyte and portal mesenchyme which allowed the efficient generation of assembloid structures, with a functional bile duct—bile canaliculi connection as we discuss later in this manuscript. By modifying this ‘self‐assembly LEGO’ approach and using human‐derived hepatocyte organoids, cholangiocyte organoids and human portal mesenchyme, the same group has generated human periportal assembloids [28]. Both mouse and human structures recapitulated *in vitro* the architectural arrangements of the *in vivo* tissue, with cholangiocyte cells surrounded by portal mesenchyme, and embedded in the hepatocyte parenchyma formed by the hepatocyte organoid. Although the full arrangement of *in vivo* portal tract structures remains absent due to the lack of portal endothelium, these landmark studies demonstrate how exploiting the self‐organization capacity of primary cells in organoid cultures can capture many aspects of liver tissue complexity *in vitro*. Interestingly, in both mouse and human models, by increasing the starting proportion of portal fibroblasts in the culture, periportal assembloids developed a fibrotic‐like phenotype, which mimicked aspects of mouse [27] and human [28] biliary fibrosis. In parallel, Chen et al. added IL17A to human CholOrgs cells to induce aspects of a biliary fibrosis phenotype [84]. Also recently, a study from the Pan and van der Laan Labs combined intrahepatic cholangiocyte organoids with various macrophages and generated structures named MaguOs: ‘macrophage‐augmented organoids’. The model, which was based on a strategy previously used to study hepatitis B virus by the Mahmoudi Lab (with overlapping authors) [85] was applied by the authors to investigate several liver‐infecting viruses, including hepatitis E, SARS‐CoV‐2 and Monkeypox [86]. Interestingly, while the authors observed SARS‐CoV‐2 infection in epithelial‐only cholangiocyte organoids, as previously reported by Zhao et al. [87], the addition of the immune compartment facilitated the identification of immune‐specific signals in viral infection of the liver [86].

### iPSC and ESC‐derived organoids

In contrast to tissue‐derived organoids, which start with one type of epithelial cell as origin, the iPSC or ESC‐derived organoids usually contain several different cell lineages co‐developing together [29, 30] (Fig. 1, bottom). Already the first iPSC liver bud model from Takebe *et al*. included unspecific niche MSC and HUVEC cells to support the development of iPSC‐hepatocyte‐like cells in culture [71], which was further improved by a follow up article where liver bud organoids were formed entirely from iPSC‐differentiated cells, with iPSC‐origin for the niche mesenchyme and endothelium [73]. A later scRNAseq analysis of iPSC‐LB performed by Camp *et al*. revealed that VEGFA produced by all liver bud cells promoted the maturation of ECs in this culture through vascular endothelial growth factor receptor 2 (VEGFR‐2), with ECs reciprocally promoting maturation of hepatoblast‐like cells [77]. Using similar protocols mimicking embryonic development, several other studies reported co‐differentiation of mesenchyme [88], mesenchyme and endothelium [89, 90], or endothelial cells in structures [91, 92], in addition to hepatoblast‐like progenitors from the same individual. Likewise, the study of ESC‐derived liver organoids described an addition of human fetal liver mesenchymal cells to the system, which improved epithelial cell differentiation and could also contribute to disease modelling in a cell autonomous manner [76]. Ouchi *et al*. achieved further cell type diversity in iPSC/ESC liver organoids by introducing Kupffer cell (KC)‐like and HSC‐like cells, using foregut‐inducing protocol followed by retinoic acid treatment and further hepatic differentiation [93]. These organoids contained hepatocyte‐like, KC‐like and HSC‐like cells and, by adding free‐fatty acid to the culture media, they could model aspects of steatosis [93], and metabolic dysfunction‐associated steatohepatitis (MASH) [94]. However, even though the cell types were simultaneously produced during the differentiation trajectory or supplemented from an embryonic‐like source, they often lacked typical tissue heterogeneity as is seen in the liver due to generally immature phenotype of iPSC‐derived organoids.

More recently, Saiki et al. described iPSC‐hepatic organoids with the introduction of liver‐specific iPSC‐derived LSECs [95]. The authors achieved LSEC differentiation, which they characterized in depth by transcriptomics, and showed the establishment of 4 different LSEC populations: arterial‐like, sinusoidal‐like and two venous‐like. By introducing air‐liquid interface culture, the authors could achieve integration of iPSC‐LSECs into hepatic organoids. The LSECs promoted the differentiation of hepatocytes into more definite subtypes, for example into hepatocytes only expressing the central vein‐specific gene GS, while some others expressed portal vein‐specific gene GLS2. The iPSC‐LSEC‐containing organoids better mimicked development by simulating positional relationship between the primitive vascular plexus and liver bud, though mature endothelial features were not fully reproduced.

In another study from that same year, Zhang *et al*. used a similar strategy to induce hepatocyte zonation [96] by introducing ESC‐derived zonated LSECs to ESC‐derived hepatocytes. The authors used PDMS micro‐well chips to co‐culture hepatocyte‐like cells with different LSECs, forming spherical organoids where ECs contacted each other to form a preliminary network. The pericentral‐like organoids could respond to CCl_4_ toxin, as they expressed higher levels of CYP2E1, while the periportal‐like organoids were more responsive to allyl alcohol, as they had more ADH activity. Each organoid showed a different response to metabolic dysfunction‐associated steatotic liver disease (MASDL)‐like conditions. In a further effort to establish a vascular network in organoids, Abilez *et al*. reported human cardiac and hepatic vascularization, modelled by organoids derived from human iPSC with reporter genes and micropatterning [97]. The authors subsequently induced mesoderm, EC and then MSC factors to co‐differentiate the cells into multi‐cell type organoids, using reporter lines to inform them about the cellular composition of the structures. Though the ECs formed a network, it was once again not perfusable and comparable to previous iPSC‐derived organoids. Finally, recently the Takebe Lab has also demonstrated the generation of fetal liver‐like organoids, which could co‐develop hepatocyte, endothelial, mesenchymal and immune lineages. Notably, the organoids contained multipotent haematopoietic cells mirroring some features of human liver development [98]. This model allowed the co‐development of hepatocyte and haematopoietic lineages and supported erythropoiesis, providing unprecedented insight into cellular cross talk in the paediatric setting.

## Mimicking organ architecture

### Engineering liver zonation

As mentioned previously, hepatocyte heterogeneity and division of labour are defined by nutrient and oxygen gradients in the liver—known as zonation [7], which also subdivides several of the liver stromal populations [18, 19, 20]. So far, while tissue‐derived organoids provide a cellular level of zonation where the hepatocyte organoid cell populations express genes specific to periportal or pericentral zonation signatures [27, 28, 69], achieving proper functional tissue zonation remains still challenging mostly owing to the difficulty of achieving micro‐modelling of engineered WNT, oxygen, hormone and nutrient gradients in the *in vitro* culture. Current models struggle to reproduce the intricate and highly nuanced zonation patterns in the microstructure of an organoid or organ‐on‐chip methods [99, 100], posing a significant engineering challenge. Nonetheless, recent advances with highly promising results have lately been published.

A recent study from the Takebe Lab described iPSC‐derived organoids which could achieve zonation at the gene expression level, in a single structure, by using two engineered iPSC lines to differentiate into hepatocytes—one overexpressing ascorbate‐producing enzyme and predisposing to periportal fate, and the other exposed to bilirubin and predisposing to pericentral fate [101]. The authors then cleverly combined the two hepatocyte organoids, by placing them in vicinity of one another and continuing the zonation‐inducing treatment. Interestingly, the organoids fused spontaneously and achieved intermediate zone in the middle, between the two induced fates, with a continuous lumen throughout the structure, and zone‐specific expression of genes in different areas. The organoids were capable of zone‐specific processing of nitrogen and glucose, as in the real tissue. The two zones of hepatocyte organoid were also differently sensitive to allyl alcohol (periportal) and acetaminophen (pericentral) [101]. While functional, the structures still lacked proper tissue architecture, likely due to the limited presence of other niche cells that normally co‐develop during differentiation—an aspect the authors did not investigate or comment on. In a complementary approach, by inducing iPSC‐derived LSEC differentiation and zonation, Takebe's group using iPSCs and Du's group using hESCs could also establish zonation signatures in hepatocytes. However, neither study achieved the simultaneous representation of both periportal and pericentral zonation in a single organoid structure [95, 96].

Also for the tissue‐derived organoids, recent studies from the Huch Lab on mouse and human periportal assembloids demonstrated that hepatocytes within these systems exhibit zonation‐related heterogeneity, encompassing both periportal and pericentral gene expression profiles [27, 28]. These assembloids, which closely recapitulate the cellular composition of liver tissue periportal region—with hepatocytes, cholangiocytes and mesenchymal cells—showed a pronounced shift towards a periportal identity compared with matched hepatocyte‐only organoids [27, 28]. This shift was characterized by increased expression of periportal markers and reduced pericentral gene signatures. In the human study, the spatial and transcriptional bias was supported by immunostaining and correlated with functional outputs (Fig. 2), as assembloids displayed enhanced periportal functions, including urea production and gluconeogenesis, alongside comparatively lower drug‐metabolizing activity. Together, these findings suggest that liver zonation is not solely governed by endothelial‐derived signals but can also be instructed by the periportal microenvironment. This highlights the critical role of multicellular interactions in establishing physiologically relevant liver zonation and function. Whether such nonendothelial, periportal instructive mechanisms operate *in vivo* remains to be determined; however, these results underscore the power of organoid systems to uncover biological principles that are difficult to resolve using traditional *in vivo* models.

### Cholangiocyte biliary tree

One crucial architectural difference of intrahepatic bile ducts compared with extrahepatic ducts is their branched morphology. Cholangiocytes in the tissue form a complex biliary tree, which contacts all the periportal areas of the liver lobules [11]. However, the original cholangiocyte organoids grew mainly as hollow lumen spheres regardless of their stem‐cell origin [39, 44, 56, 74, 75], with some studies reporting spontaneous emergence of rare tube‐like structures [74, 75]. The first study to attempt the formation of cholangiocyte tubes did so by populating collagen‐densified structures with human adult extrahepatic cholangiocyte organoids. The work demonstrated proof‐of‐principle for generating transplantable bioengineered bile duct tissue, with potential applications in the treatment of common bile duct diseases [58]. Several years later, by adapting the original protocol to grow human cholangiocyte organoids, Roos et al. presented the first branched cholangiocyte organoid model [102]. This change in morphology occurred in about 50% of organoid structures and could be maintained after passaging, though interestingly it could not be achieved in extrahepatic or embryonic organoids. The authors were able to achieve higher cholangiocyte heterogeneity as in tissue and showed increased functionality of the structures in terms of their transporter‐mediated features.

### Hepatocyte bile canaliculi networks

Hepatocytes contribute to the liver architecture by forming bile canaliculi, in addition to performing different regenerative and metabolic functions. In iPSC‐derived organoids, two studies showed bile canaliculi structures using electron microscopy [103, 104]. Both studies also stain the structures for apical domain markers, but bile canaliculi appeared as circular lumens rather than channels in those organoids [103, 104]. Shinozawa et al. reported fluorescein transport assay, used to show the successful transport of fluorescent bile acid analogues into the lumen of bile canaliculi. The organoids presented good uptake of the dye into hepatocytes; however, they showed no clear emergence of bile canaliculi networks [104].

While several studies have reported the expression of apical domain marker genes in hepatocyte organoids, the first human fetal HepOrgs were unique in demonstrating the formation of bile canaliculi between several hepatocytes, as evidenced by whole‐mount staining for the canalicular transporter MRP2. However, these bile canaliculi culminated into cystic structures within the grape‐shaped organoids [52]. The parallel paper by Peng *et al*. provided CD26 staining in murine organoids, which was enriched in bile canaliculi [57]. However, the presence of bile canaliculi structures within those HepOrgs was hard to evaluate as the CD26 staining was also visible on the basolateral domain of hepatocytes [57]. Even though the hepatocytes could uptake bile analogue and transport it to their apical domain, only small spherical bile canaliculi between a few neighbouring hepatocytes were visualized, as opposed to a full bile canaliculi network [57].

The improved human fetal organoids, reported by Hendricks et al., showed much more homogenous MRP2 staining, indicative of bile canaliculi formation. Nevertheless, bile canaliculi network formation was not reported [66]. A year later, another study provided the first comprehensive analysis of bile canalicular network in improved murine HepOrgs [27]. These networks were functional, and hepatocytes could metabolise and transport several different bile acid analogues and fluorescent phosphatidylcholine to bile canaliculi. Moreover, the diameter of achieved structures was close to that observed in tissue, without the emergence of cholestatic features. The same year, human HHO and h‐HepOrgs from Igarashi et al. and Yuan et al., respectively, were also shown to form bile canaliculi [28, 69]. These structures were functional and, in the patients studied by Yuan *et al*., formed a network that resembled the *in vivo* tissue, albeit with some variation.

### Hepatocyte bile canaliculi – Bile duct connection

Another important architectural feature of the liver is the establishment of cholangiocyte ducts within the hepatocyte parenchyma, and the connection between the hepatocyte bile canaliculi and bile duct, as seen in the canal of Hering [6]. For the iPSC‐derived organoids, already the early models showed co‐development of hepatocyte‐like and cholangiocyte‐like cells, albeit without correct architectural arrangement [71, 77, 89]. To establish the connection between epithelial cell types, Ramli *et al*. used a combination of defined chemical factors and changing media regimes, which directed human iPSCs through the stages of definitive endoderm, posterior foregut, hepatic endoderm and hepatoblasts to organoid structures [103]. Formed structures consisted of a hepatocyte‐like cell core with cystic structures formed by cholangiocyte‐like cells attached on the outside of the structure. However, no bile canaliculi‐bile duct connections were visualized [103].

The modelling of the bile canaliculi—bile duct connection was addressed later by the Huch Lab, in a study which reported ‘periportal assembloids’ as discussed in the section ‘improving complexity’. These assembloids were generated by combining improved hepatocyte organoids with cholangiocytes and portal mesenchyme, resulting in a functional three cell type structure. Hepatocytes formed a pseudo‐parenchyma, within which the cholangiocytes formed a small lumen, connected to the hepatocyte bile canaliculi, and were surrounded by portal mesenchymal cells. An incredible feat of organoid engineering, these structures were able to transport bile from the bile canaliculi to the bile duct in timeframes comparable to the real tissue [27]. Similar structural features were observed in human periportal assembloids, although the assessment of functional connection was not reported [28].

### Formation of perfusable blood vessels

Many of the iPSC‐organoids are co‐derived with HUVEC or iPSC‐ECs [71, 73], and some adult‐derived organoids also report co‐culture with HUVECs [52]. While studies claim the emergence of an EC network, none to date have shown proper vessel formation, nor was the EC network shown to be perfused. The establishment of perfusable endothelial vessel‐containing liver organoids will constitute a critical step in the future development of liver and other organoid systems and could provide better differentiation and long‐term maintenance of organoids, removing the typical necrotic core seen in bigger organoid structures.

## Recapitulating cancer complexity in a dish

### Cancer cells

First primary liver cancer (PLC) organoids, also known as tumoroids, were derived from hepatocellular carcinoma (HCC), cholangiocarcinoma (CC) or combined HCC/CC (CHC) tumour resection samples by the Huch Lab [105]. While containing only the epithelial compartment, the organoid models very closely recapitulated the unique features of each of the liver cancer subtypes of patients, mimicking gene expression and mutational landscape *in vitro*. Tumoroids expanded long term, and the authors demonstrated principles of drug testing and biomarker identification in their study. Interestingly, well differentiated HCC could not be expanded in culture similarly to hepatocytes at the time of this study [105]. A year later another study, this time from the Heim Lab, used needle biopsy samples to establish PLC tumoroids from HCC and CC samples [106]. Needle biopsies allowed the establishment of tumoroids not only from resection stages, but from all stages of tumour progression, and similarly to the first study, the authors showed good agreement of the tumoroids with the clinical samples regarding their gene expression and genetic structure. Later, another study showed improvement of cholangiocarcinoma modelling by inducing branching in the cancer context [102]. The tumoroids were proliferative in the tips and branches, and showed increased drug resistance to combination therapy compared with luminal spherical CC organoid lines [102].

Tumoroids for bile duct ampullary adenocarcinoma [107], gallbladder cancer, neuroendocrine carcinoma of the ampulla of Vater [108] and childhood cancer hepatoblastoma [109] have also been reported. In addition to the drug tests in the first liver tumoroid publications, the models have been extensively used for drug screening [110, 111, 112, 113, 114]. Toxicity comparison between healthy cholangiocyte organoids and cancer organoids was also elegantly demonstrated in several studies [108, 109], potentially identifying novel therapeutic avenues without affecting the healthy tissue. Importantly, the models recapitulated intratumor and intertumoral heterogeneity of cells within one patient and between patients.

An additional advantage of organoid models is their ease of manipulation. Artegiani et al. generated a BAP1 protein knockout (KO) in human liver adult cholangiocyte organoids to investigate mechanisms of cholangiocarcinoma emergence through changes in chromatin induced by this mutation [115]. The BAP1‐KO organoids developed a darker morphology and proliferated faster, exhibited unusual motility, and loss of typical cholangiocyte cell polarity. In the background of classical cancer mutations (TP53, PTEN, SMAD4 and NF1), BAP1 deletion caused the emergence of CC upon xenotransplantation in mice. Using a similar approach, another study reported murine liver tumoroids for the modelling of liver cancer—this time genetically engineering cholangiocyte organoids using a combination of RNAi, Cre and CRISPR systems [116]. The authors introduced KRAS and TP53 mutations to induce a cholangiocarcinoma‐like phenotype and a TP53 mutation with oncogenic MYC combination. The authors also could achieve a tumour type similar to HCC upon transplantation in mice.

### Tumour microenvironment

The tumour is not only composed of the transformed epithelium—it also contains fibroblasts, blood vessels, immune cells and ECM, which all play a role in the development of cancerous lesions [117, 118]. Therefore, the introduction of tumour microenvironment was the next step in the development of complexity in the cancer organoid models. To this end, one group established murine liver cancer organoids derived from tumours induced by the carcinogen N‐nitrosodiethylamine (DEN) [119]. The emergence of tumours in mice was variable, and the tumoroids were also variable in morphology and cell of origin. The authors stated that while this mouse model did not fully recapitulate liver cancer in human patients, it could provide important insights into the mechanisms of cancer emergence. Based on this study, the same group expanded on the model to introduce cancer‐associated fibroblasts (CAFs) to both murine‐derived and patient‐derived tumoroids [120]. Isolated CAFs formed a net around tumoroids and promoted their proliferation, at least partially through paracrine signalling. Conditioned media from tumoroids reciprocally increased the numbers of CAFs, in both mouse and human, while co‐injection into mice of CAF‐supplemented tumoroids resulted in more tumour formation efficiency and increased tumour growth. Finally, the authors showed that CAFs protected tumour organoids from anticancer treatment once again, at least in part through paracrine signalling [120]. Another study published in the same year recapitulated the aspects of tumour microenvironment by supplying CAF‐derived signals via conditioned media from fibroblasts or HSCs producing a ligand important in the cancer microenvironment. Exposure to these factors promoted HCC growth and increased the metastatic potential in patient‐derived tumoroids [121].

In the same study which reported a rare bile duct ampullary adenocarcinoma culture [107], the authors could maintain patient‐derived tumoroids and some of the patient immune and mesenchymal cell repertoire on air‐liquid interface, with a specific focus on T cells—many of which could be maintained for 60 days in culture by the addition of IL‐2 to the culture media. However, while the samples could be passaged, they lost typical cancer morphology and stroma association with time. In most of the samples originally containing myofibroblasts, myofibroblasts were progressively lost. In a few selected cases, conversely, myofibroblasts overgrew the tumoroids [107]. Two other studies introduced engineered T cells to the co‐culture with HCC tumoroids, to model immunotherapy response rather than improving modelling of tumour microenvironment. In the first study, the authors confirmed the expression of chronic hepatitis B virus (HBV) surface proteins on many of the HCC samples, and engineered CAR T cells to selectively target that cancer population, a T‐cell population later characterized by high expression of CD39 surface marker [122]. In another study, the authors coated T cells with flexible DNA networks to protect them from premature cell death and therefore improved their therapeutic potential, showing higher activation and killing ability of those T cells against HCC tumoroids [123]. In both studies, the T cells were simply added to tumoroids after their extraction from Matrigel, and co‐cultures were then only kept for 24 h, which induced cell death in tumoroids [122, 123]. Additionally, cellular interactions were not compared with the native tumour tissue.

## Current limitations and future directions

Despite the impressive improvements in the past decade, still many caveats exist in both iPSC/ESC and tissue‐derived organoids. The iPSC/ESC‐derived organoids remain immature, resembling embryonic stages of liver development. Moreover, the cell ratios of these organoids cannot be controlled. The challenges lie in the further differentiation and dissociation/reassociation of these organoids into correct cell ratios and organ architecture. On the contrary, the adult tissue‐derived organoids are more differentiated, and besides the recent periportal assembloids models often only consist of one epithelial cell type, the addition of each extra cell type is challenging. The method to isolate correct primary cell populations and maintain their phenotype in culture, combine them with organoids and find the suitable media that supports all cell types remains elusive for most human organs, and therefore, these organoids remain limited in the cell types they contain. Both the iPSC/ESC and tissue‐derived organoids are summarized in Table 1. Currently, all organoids are too small to establish an axis of oxygen and metabolite gradient to fully model liver lobule. Some progress has been made using the so‐called ‘organs‐on‐chip’ to solve this issue, reviewed elsewhere [99, 100]. Nevertheless, those models also fail to recapitulate the complexity and architecture of the liver lobule and cannot be cultured long term.

**Table 1:** Concise comparison table of the main characteristics of induced pluripotent stem cells (iPSC)/embryonic stem cells (ESC) and tissue‐derived organoids.

Characteristic	iPSC/ESC	Tissue‐derived
Maturity	Fetal‐like	Adult‐like
Expansion potential	High (as iPSC), low when ‘organoid’	High at organoid stage
Cell type complexity	High, but uncontrolled	Low, but controlled
Genetic stability	Low at the iPSC stage	High
Zonation	Partial zonation on organoid‐level	Partial zonation on organoid‐level
Best suitability	Cell therapy, disease modelling for developmental disorders, development modelling	Disease modelling, personalized medicine, study of adult tissue biology, homeostasis and repair

Also, defining functional maturity of liver organoid models remains challenging. Hepatocyte function is often inferred from transcriptomic similarity to PHHs, but gene expression alone does not capture the quantitative and dynamic aspects of metabolic activity, even though it is generally assessed in all the studies (Fig. 2). Notably, cytochrome P450 (CYP) activity in organoids often remains lower or less stable than in freshly isolated PHHs, underscoring the need for standardized functional benchmarking. In addition, long‐term culture of liver organoids can lead to metabolic rewiring, including lipid dysregulation, reflecting adaptation to the *in vitro* conditions rather than true physiological maturity. Recent advances in multicellular systems, such as liver assembloids, further suggest that hepatocyte specialization is strongly influenced by the microenvironment (e.g., [28]). As discussed, these models exhibit zonation‐related heterogeneity and functionally distinct hepatocyte states, highlighting that hepatocyte maturity is context‐dependent and shaped by cellular interactions and spatial organization.

While many authors comment on faithful recapitulation of tissue architecture—it is rarely the case. The most important missing component in all organoid models is currently the absence of fully formed endothelial cell networks, containing veins and arteries. We are yet to observe a perfused liver organoid, which incorporates all the different endothelial populations in a continuous blood‐containing vein‐like network, capable of transporting oxygen and nutrients. Perfusion of organoids will be important not only for the interactions between different cells and endothelium, but also for the delivery of nutrients to the centre of complex organoids, which remain quite dense and can develop a necrotic core with larger structures. Furthermore, the complexity of immune cells in organoids is far from those observed in tissues. Tissue‐resident T cells were recently introduced in human intestinal organoids [124], and similar approaches can be used to improve resident immune population modelling in other organs. However, for the liver, contrary to the intestine, it will be important to take into account that immune liver‐resident cells reside in specialized niches and reproducing this specialization will be critical if we aim to achieve proper tissue architecture. Finally, the lack of proper microenvironment and full maturation of the epithelial cells is currently the reason, in addition to high costs, why human organoid biobanks are not used more often to perform drug screening for personalized medicine.

In the long term, liver organoids are also the hope to replace liver transplants, and instead growing matched mini‐livers for the patients would solve the scarcity of tissue and immune rejection of currently used transplantation methods. However, the majority of organoid approaches use Matrigel, or similar ECM derivatives, which are sourced from mouse sarcoma cells and are unsuitable for human usage [125]. Also, many of the other cytokine components and media are currently produced using animal derivatives and are unsuitable for transplantation [126, 127]. Especially regarding iPSC cells, special care must be taken that upon potential transplantation, those cells do not spontaneously form tumours [128, 129]. The final stage in the modelling of liver physiology and disease would be the connection with other organs and co‐derivation of several organs in one structure. So far, only one report describes a hepatic, biliary and pancreatic structure, derived together from iPSC and modelling multiorgan system development in a dish—the first study of human endoderm organogenesis with organoids at this scale [130]. Future research will aim at improving inter‐organ communication in a dish and modelling of this communication in disease.

## Conclusions and future perspective for the liver organoid field

The organoid field is rapidly transitioning from infancy to a phase of transformative maturity. As noted in previous reviews [70], the past decade has seen an exponential rise in liver organoid studies, paralleled by steady advances in cellular diversity and the architectural sophistication of these 3D systems. Yet, what has been achieved so far likely represents only a glimpse of their full potential.

We foresee liver organoids emerging as a central platform to decode fundamental principles of human liver biology. Beyond modelling development, these systems will enable precise interrogation of species‐specific traits that cannot be captured in traditional animal models, redefining how we can bridge basic and human biology. At the same time, liver organoids are poised to reshape translational research, serving as powerful engines for toxicology screening, drug discovery, regenerative medicine and truly individualized therapeutic strategies.

Looking ahead, the convergence of developmental biology, bioengineering and rapidly evolving AI‐driven approaches will propel the field into a new era. Future liver organoids are expected to transcend current limitations, achieving unprecedented levels of fidelity—not only in function and cellular composition, but also in spatial organization, multicellular interactions and dynamic tissue behaviour. In this vision, organoids will not merely model the liver; they will become predictive, tuneable and ultimately indispensable systems that redefine both our understanding of the organ and our ability to treat its diseases.

## Conflict of interest

MH is an inventor in several patents on organoid technology. MH and AMD are inventors in a patent on human liver assembloids.

## Author contributions

MH and AMD designed the project and wrote the paper, contributing equally.
